# Use of the activPAL^®^ triaxial accelerometer to estimate total energy expenditure in low-income women: differences between body mass index classifications

**DOI:** 10.20945/2359-3997000000616

**Published:** 2023-05-29

**Authors:** Mateus de Lima Macena, Déborah Tenório da Costa Paula, André Eduardo da Silva, Dafiny Rodrigues Silva Praxedes, Karina Pfrimer, Eduardo Ferriolli, Telma Maria de Menezes Toledo Florêncio, Nassib Bezerra Bueno

**Affiliations:** 1 Universidade Federal de Alagoas Faculdade de Nutrição Maceió AL Brasil Faculdade de Nutrição, Universidade Federal de Alagoas, Maceió, AL, Brasil; 2 Universidade Federal de São Paulo Escola Paulista de Medicina São Paulo SP Brasil Escola Paulista de Medicina, Universidade Federal de São Paulo, São Paulo, SP, Brasil; 3 Universidade de São Paulo Faculdade de Medicina de Ribeirão Preto Ribeirão Preto SP Brasil Faculdade de Medicina de Ribeirão Preto, Universidade de São Paulo, Ribeirão Preto, SP, Brasil

**Keywords:** Accelerometry, doubly labeled water, energy expenditure, obesity, physical activity

## Abstract

**Objective:**

This study aimed to assess the agreement between the total energy expenditure (TEE) estimated by the activPAL^®^ triaxial accelerometers (ACC) and the TEE measured by the doubly labeled water method (DLW), as well as to assess if these values differ between the classifications of body mass index (BMI).

**Materials and methods:**

This is a cross-sectional study. Low-income adult women (19-45y) with BMI ≥ 18.5 kg/m^2^ were included. Accelerometry data (activPAL^®^) were collected over 7 consecutive days, which were used to calculate TEE-ACC and compared with DLW data. The Bland-Altman method, concordance correlation coefficient and root mean square error were used to assess agreement between methods.

**Results:**

The sample consisted of 55 women with a mean age of 31 ± 5 years. The agreement between TEE-ACC and TEE-DLW showed a bias of -142.5 kcal (-7.1%). Among the BMI classifications, participants with normal weight show a bias of -417.1 kcal (-21.0%), participants with overweight, -87.5 kcal (-3.9%) and participants with obesity, 97.5 kcal (4.3%). Furthermore, the bias between the methods showed a significant and positive correlation with the body weight (r = 0.49; p < 0.01).

**Conclusion:**

The TEE-ACC estimates from activPAL^®^ were reasonably accurate when compared to the TEE-DLW, especially in women with overweight and obesity, being much less accurate in individuals with normal weight.

## INTRODUCTION

Knowing the energy needs of populations through estimates or measurements of total energy expenditure (TEE) is essential for establishing and monitoring strategies for the prevention and treatment related to body weight control (1-3). The TEE of adult individuals is the result of the sum of their basal metabolic rate (BMR), the thermic effect of food, and physical activity energy expenditure, with the latter being the most variable and widely researched (4,5). Several methods have been developed to assess TEE, and the technique of doubly labeled water (DLW) is considered the gold standard for measuring TEE. However, DLW has high and unfeasible costs for clinical practice (6,7). Alternatively, the factorial method (i.e., multiplying the BMR by the physical activity level [PAL]) with the aid of predictive equations of energy expenditure is often applied for these estimates. However, the equations still show variable results, which may impair the effectiveness of the clinical strategies. Furthermore, studies evaluating the validity of these equations for estimating TEE are scarce (1).

Part of the failure to estimate the TEE using predictive equations may be related to difficulties in measuring PAL. Currently, numerous self-reported questionnaires are available for PAL estimation, such as the International Physical Activity Questionnaire (IPAQ), which is considered a practical and fast method and a cost-effective tool (8). Although the application of several questionnaires has been validated for estimating the PAL of individuals, low-income populations have important limitations regarding their use (9). Individuals of low socioeconomic class often present low schooling or are illiterate, which can interfere with the possibility of application and interpretation of the questions contained in the questionnaires, generating under-or overestimation of the activities performed. Hence, the estimation of TEE in the low-income population using the factorial method may be compromised.

Therefore, the search for methods for estimating TEE that is easy to apply and more accessible is constant. Accelerometers has been gaining interest from the scientific community because they are non-invasive, cheaper than the DLW technique, easy to handle, and capable of effectively measuring the PAL of individuals, with decreased chances of recall bias (10,11). This equipment has also been used to estimate TEE based on determining the metabolic equivalent of task (MET) (12). However, the accuracy of the TEE values provided by accelerometers compared to those measured by methods considered the gold standard is variable (13,14). In the study by Johannsen and cols. (15), for example, the activity monitors SenseWear Pro3, and SenseWear mini showed discrepancies of 4% and <0.01% in TEE estimates, respectively, whereas in the study by Brazeau and cols. (16), the SenseWear Armband had a percentage difference of 12.4%. Incorrect TEE measurements can lead to inaccurate interventions, making studies that aim to validate TEE estimation essential.

Systematic reviews have already shown that physical activity monitors are somewhat effective in estimating TEE, but biological measures such as heart rate are still needed to improve its accuracy (10). However, few studies have evaluated this theme in low-income populations. Furthermore, we are not aware of studies that evaluated the agreement between the accelerometry and DLW methods according to the classification of body mass index (BMI), since the studies available in the literature do not distinguish weight status (normal weight, overweight, obesity) in their analyzes (10,11). It is still questioned whether the supposed longer time of individuals with obesity in sedentary or light activities may bring some discrepancy in the agreement in the measurements of TEE by accelerometry when compared to DLW (17). Furthermore, given the different types of accelerometers available, investigations on specific models are welcome to enhance our understanding in this scenario. Hence, this study aimed to assess the agreement between the TEE estimated by the activPAL^®^ triaxial accelerometer (TEE-ACC) and the TEE measured by the DLW method (TEE-DLW) in low-income women as well as to assess whether these values differ between the BMI classifications.

## MATERIALS AND METHODS

### Ethical aspects

This study was conducted in accordance with the recommendations of the Declaration of Helsinki, and all procedures were approved by the Research and Teaching Ethics Committee of *Centro Universitário Cesmac* under protocol 1588/12. The approval of the project of the present study precedes the establishment of Plataforma Brasil, therefore, it does not present a Certificate of Presentation for Ethical Appreciation. All participants were informed about the study, agreed to participate in the research, and signed a written consent form.

### Study location and population

Sampling was non-probabilistic, and participants were recruited at the Center for Nutritional Recovery and Education (CREN). CREN is located in the region with the lowest human development index in Maceió-Alagoas, Brazil. This center treats chronically malnourished children and their mothers and caregivers from more than 24 slums in the municipality.

The present study included adult women aged between 19 and 45 with a BMI ≥ 18.5 kg/m^2^. Women who were pregnant, breastfeeding, had any physical condition that precluded anthropometry, and had an unstable self-reported body weight in the last month or during the TEE collection period were excluded. Women who used chronic medications (antidiabetic, diuretic, or thyroid hormone replacement) were also excluded.

### Anthropometric data

Body weight was measured using a digital scale (Filizola, São Paulo, Brazil), and height was measured using a portable stadiometer. The BMI was calculated and categorized as recommended by the World Health Organization: normal weight (18.5 kg/m^2^ ≤ BMI ≤ 24.99 kg/m^2^), overweight (25.00 kg/m^2^ ≤ BMI ≤ 29.99 kg/m^2^), and obesity (BMI ≥ 30.00 kg/m^2^) (18).

### Measurement of total energy expenditure

The participants’ TEE was measured using the DLW (^2^H_2_^18^O) multiple-point technique, in which all participants received a single dose of DLW. The dose received considered the total body water of the women included and was composed of 0.12 g of water labeled with 99.8% H and 2 g of 10% water labeled with ^18^O/kg of estimated body water. Body water was assumed to represent 50% of the women’s body weight (19). Urine samples were collected from each participant on the 1st, 2nd, 3rd, 7th, 12th, 13th, and 14th days after dose administration. These samples were used to compare the enrichment of the baseline sample, which is necessary for calculating isotopic dilution spaces and TEE. Individuals were weighed on the first and last days of measurement, and only those with a variation of <1% of the body weight were analyzed. The enrichment of samples collected 14 days after dosing was used to evaluate the elimination rates of ^2^H and ^18^O, calculated according to Speakman (20). A mean respiratory quotient of 0.85 was assumed to calculate TEE. The isotopic analysis of the samples by mass spectrometry isotopic ratio for 18O (ANCA 20-20; Europe Scientific) and 2H (ANCA 20-22; Sercon) was performed at the Laboratory of Mass Spectrometry of the *Faculdade de Medicina de Ribeirão Preto*, accredited by the International Atomic Energy Agency.

### Measurement of body composition

The body composition was determined using formulas to obtain the dilution spaces in the water of the stable isotopes (^2^H) in relation to the basal values (21). The determination of the fat-free mass (FFM) considered the hydration constant of this tissue to be 73.2% water. Body fat (BF) was obtained by subtracting FFM from body weight (21).

### Accelerometry data

Triaxial accelerometers (activPAL^®^, activPAL™ 3C, PAL Technologies Ltd, Glasgow, UK) were first used to estimate PAL and were later used to estimate TEE. Participants wore this equipment affixed to the anterior portion of the right thigh for seven consecutive days (168 h). The accelerometer was affixed with the aid of a hypoallergenic adhesive (TegaDerm, 3M) on the dependencies of the CREN during the first 7 days of the DLW assessment. This could only be removed by a researcher, so, if the participant reported any discomfort, she should return to CREN so that a researcher could change the adhesive. However, no major issues were reported. Therefore, all participants included in the present study had 168h accelerometer data. In addition, participants were advised not to perform water activities, such as swimming in the pool or in the beach.

Data were analyzed using the activPAL3™ software (v7.2.32, PAL Technologies Ltd), which calculates the time subjects spend sitting, lying, standing, and walking every tenth of a second and then provides an estimate of MET for the entire period of use based on default values for sitting/lying down (1.25 MET), standing (1.40 MET), and walking at a cadence of 120 steps per minute (4.00 MET). Accelerometer data analysis software provided the MET value for the entire period that the subjects used by multiplying the MET value of each activity by the duration of the activity. For cadences that differ from 120 steps per min, the following equation was used to calculate the MET estimate: MET.h = (1.40 × d) + (4.00 – 1.400) × (c/120) × d, where c is the cadence (steps per minute) and d is the duration of the activity (in h). According to the Food and Agriculture Organization (4), the MET value obtained by accelerometer analysis is closely related to the physical activity ratio, which is used to estimate an individual’s PAL when using physical activity questionnaires. Thus, the amount of MET was divided by 168h (time of accelerometer use) to obtain the PAL per hour for each participant. Using the premise that 1 MET is equivalent to 3.5 mL of oxygen/kg/min or 1 kcal/kg/h (22), to estimate TEE using PAL, the PAL estimated by the accelerometers was multiplied by the body weight, and for 24 hours.

This way of calculating TEE is based on the assumptions reported by Ainsworth and cols. (22), since it is known that the energy expenditure of an individual at rest would be equivalent to 1 MET (1 kcal/kg/h), and the other activities would vary according to the estimated times performing the different types of activity, whether performed sitting/lying down, standing or walking, it is plausible that this form can cover all the components of the TEE (resting and sleeping energy expenditure, activity energy expenditure etc.).

#### Factorial model

To make comparisons with the TEE calculated by the activPAL, we also calculated the TEE using the factorial method (BMR calculated by predictive equations x PAL). To this, the predictive equation proposed by FAO/WHO/UNU (1985) was used, which can be observed below:

- 18-30 years: (14.7 x weigth) + 496;
- 30-60 years: (8.7 x weigth) + 829.

This predictive equation showed the lowest bias in individuals with overweight and obesity compared to the indirect calorimetry method, among 47 equations evaluated in the systematic review by Macena and cols. (1).

### Statistical analysis

Categorical variables are presented as relative and absolute frequencies, whereas continuous variables are presented as means and standard deviations. Data were separated into BMI classification groups (normal weight, overweight, and obesity). To test the differences in continuous variables between the groups, a one-way ANOVA test was used, and later, the Bonferroni correction was applied to determine which groups showed differences.

To evaluate the methods of agreement between TEE-DLW and TEE-ACC, three different methods were used: (I) The Bland-Altman method was used both using the gross differences (kcal), both with the percentage differences, to reduce the proportionality bias of the analyzes (23). The limits of agreement and their respective 95% confidence intervals (95% CI) were calculated to assess whether the TEE-ACC did not present a significant bias in relation to the TEE-DLW and the paired sample t-test between the TEE-ACC and the TEE-DLW. (II) The root-mean-square error between TEE-ACC and TEE-DLW was calculated, where smaller values represent a better agreement between the methods. (III) The concordance correlation coefficient (CCC), which considers both precision (Pearson’s correlation coefficient) and accuracy (using a bias correction factor that measures the deviation of the best-fit line from the 45 °line), was estimated for each pair of measurements (24). Furthermore, the standardized bias between the methods and precision, defined as the prevalence of individuals with TEE-ACC within an adequate range (±10%) of the TEE-DLW, were calculated.

Pearson’s correlation was also used to measure whether the bias between the methods correlated with body weight (kg). Finally, to explore whether there was any relationship between PAL and its markers (sitting/lying time (h/d), standing time (h/d), and walking time (h/d)) and the calculated bias (kcal) between TEE-ACC and TEE-DLW, Pearson and Spearman’s correlations were performed. All analyses were performed using the statistical software MedCalc version 18.11.3 (MedCalc Software Bvba), in which an alpha value of 5% was adopted.

## RESULTS

The sample consisted of 55 women with a mean age of 31 ± 5 years, with 30.9% (n = 17) classified as having normal weight, 43.6% (n = 24) as overweight, and 25.5% (n = 14) as with obesity. Table 1 shows the general characteristics of the sample as well as the TEE-DLW and TEE-ACC for each BMI classification.

**Table 1:** Sample characteristics

Variables	All sample (n = 55)		Body mass index classifications						p-value*
			Normal weight (n = 17)		Overweight (n = 24)		Obesity (n = 14)		
	Mean	SD	Mean	SD	Mean	SD	Mean	SD	
Age (years)	31.04	5.42	31.06^a^	6.40	30.88^a^	5.24	31.29^a^	4.79	0.97
Body weight (kg)	66.47	12.26	54.24^a^	8.09	67.30^b^	5.66	79.92^c^	9.81	<0.01
Height (m)	1.55	0.07	1.55^a^	0.08	1.56^a^	0.06	1.55^a^	0.09	0.96
Body mass index (kg/m^2^)	27.36	4.49	22.28^a^	2.16	27.63^b^	1.41	33.07^c^	2.35	<0.01
Body fat (%)	41.98	5.93	36.23^a^	6.71	43.07^b^	2.49	47.08^c^	2.51	<0.01
Metabolic equivalent task/hour	1.47	0.06	1.47^a^	0.07	1.46^a^	0.05	1.48^a^	0.06	0.43
Sitting/lying down time (h/d)	15.07	1.81	15.06^a^	1.93	15.57^a^	1.56	14.20^a^	1.88	0.78
Standing time (h/d)	6.48	1.51	6.43^a^	1.35	6.10^a^	1.36	7.19^a^	1.79	0.09
Walking time (h/d)	2.44	0.75	2.50^a^	0.87	2.31^a^	0.63	2.61^a^	0.81	0.49
TEE-DLW	2118.05	361.75	2032.68^a^	490.89	2071.21^a^	237.16	2302.04^a^	312.15	0.08
TEE-Accelerometry	1975.52	370.06	1614.85^a^	259.04	1983.66^b^	146.55	2399.51^c^	290.67	<0.01
TEE-Factorial method	2114.61	220.62	1915.12^a^	186.65	2119.06^b^	96.86	2349.21^c^	177.08	<0.01

DLW: doubly labeled water; TEE: total energy expenditure.

The different superscript letters characterize means with statistically significant differences between the groups of classifications of body mass index by the Bonferroni test.

* p-value for the One-Way ANOVA test.

**Figure 1:**
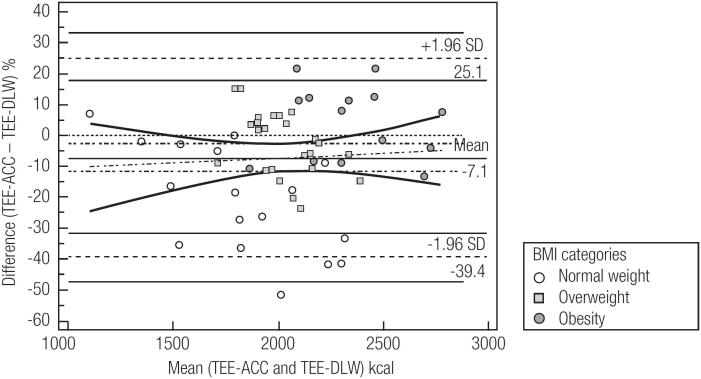
Bland and Altman plot for total energy expenditure estimated by accelerometer and measured by doubly labelled water.

**Figure 2:**
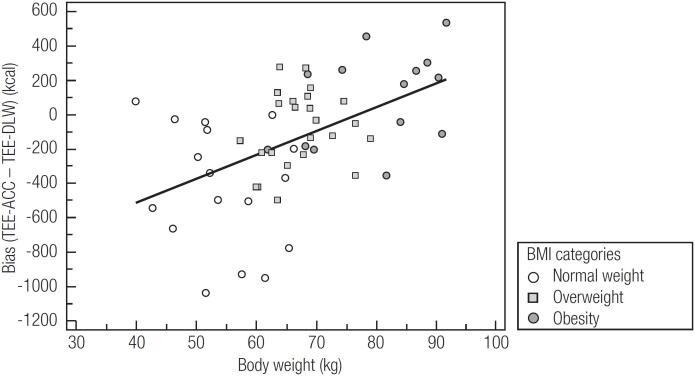
Relationship between bias (total energy expenditure estimated by accelerometers and measured by doubly labelled water) and body weight.

The assessment of agreement between the TEE-ACC and TEE-DLW with the overall sample and by BMI classification is shown in Table 2 and Figure 1. Considering the entire sample, there was a bias of -142.5 kcal (-7.1%) between TEE-ACC and TEE-DLW, and a CCC of 0.52. Among the BMI classifications, overweight participants had the best estimate of the TEE-ACC compared to the TEE-DLW (bias [kcal] = -87.5; bias [%] = -3.9; limits of agreement [%] = -18.9; 27.5; root mean square [kcal] = 230.6; CCC = 0.35). The worst TEE-ACC estimate was observed in the group of individuals with normal weight (bias [kcal] = -417.1; bias [%] = -21.0; limits of agreement [%] = -54.9; 12.9; root mean square [kcal] = 543.5; and CCC = 0.36). Furthermore, the bias between methods showed a significant positive correlation with body weight (r = 0.49; p < 0.01; Figure 2).

**Table 2:** Evaluation of the agreement between the total energy expenditure estimated by accelerometry (activPAL) or factorial method and the total energy expenditure measured by doubly labeled water in the classifications of body mass index

Body mass index classifications	Root-mean-square error (kcal)	Bias (kcal)^1^	LoA	Bias (%)^2^	LoA	LoA LL	LoA UL	t test	Standardized difference	Concordance correlation coefficient	Maximum positive error (%)	Maximum negative error (%)	Precision (%)^4^
			LL; UL (kcal)		LL; UL (%)	95% CI	95% CI	p-value^3^					
**activPAL**													
All sample (n = 55)	367.1	-142.5	-811.7; 526.6	-7.1	-39.3; 25.1	-47.0; -31.7	17.4; 32.7	0.34	-0.42	0.52	24.47	-41.01	47.3
Normal weight (n = 17)	543.5	-427.1	-1120.2; 284.5	-21.0	-54.9; 12.9	-70.4; -39.4	-2.5; 28.4	0.08	-1.16	0.36	7.30	-41.01	35.3
Overweight (n = 24)	230.6	-87.5	-514.7; 339.6	-3.9	-10.7; 17.1	-32.9; -17.1	9.2; 25.0	0.03	-0.40	0.35	16.34	-21.14	58.3
Obesity (n = 14)	281.9	97.5	-440.7; 635.6	4.3	-18.9; 27.5	-30.9; -7.0	15.5; 39.4	0.67	0.35	0.55	24.47	-12.27	42.9
**Factorial method**													
All sample (n = 55)	315.2	-3.4	-627.0; 620.1	0.8	-29.1; 30.8	-36.2; -22.0	23.73; 37.97	<0.01	-0.01	0.43	56.8	-29.1	45.4
Normal weight (n = 17)	405.6	-117.5	-901.9; 666.8	-3.4	-44.1; 37.4	-62.8; -25.5	18.7; 56.0	<0.01	-0.29	0.39	56.8	-29.1	35.2
Overweight (n = 24)	231.4	47.9	-405.62; 501.33	2.8	-19.0; 24.6	-27.1; -10.8	16.4; 32.7	<0.01	0.20	0.17	26.3	-16.8	54.1
Obesity (n = 14)	314.2	47.2	-584.8; 679.1	2.6	-24.2; 29.4	-38.1; -10.4	15.5; 43.2	0.06	0.14	0.18	30.0	-17.8	42.8

LL: lower limit; LoA: limits of agreement; TEE: total energy expenditure; UL: upper limit.

1 Difference between TEE-ACC or factorial method and TEE-DLW.

2 Percentage mean of the difference between TEE-ACC or factorial method and TEE-DLW.

3 p value for a t test for paired samples, comparing the mean TEE-ACC or factorial method with the mean TEE-DLW.

4 Prevalence of individuals who present total energy expenditure estimated by accelerometry or factorial method that fall within an adequate range (±10%) of the total energy expenditure measured by doubly labeled water.

The factorial method (BMR x PAL) produced a lower bias in relation to the DLW method (-bias [kcal] = -3.4; bias [%] = 0.8), however, the prevalence of individuals who presented a variation between ± 10% was lower (45.4%), as can be seen in Table 2.

Finally, exploratory analyses evaluating the influence of PAL, sitting/lying down time, standing time, and walking time showed no significant correlation between TEE-ACC and TEE-DLW (Table 3).

**Table 3:** Exploratory analysis of the influence of physical activity level, sitting/lying down time, standing time and walking time on the bias between energy expenditure estimated by accelerometry and energy expenditure measured by doubly labeled water

Body mass index classifications	Physical activity level		Sitting/lying down time (h/d)		Standing time (h/d)		Walking time (h/d)	
	rho^1^	p-value	r^2^	p-value	r^2^	p-value	r^2^	p-value
All sample (n = 55)	-0.05	0.69	0.01	0.96	0.02	0.83	-0.06	0.62
Normal weight (n = 17)	-0.25	0.33	0.16	0.52	-0.17	0.50	-0.08	0.73
Overweight (n = 24)	-0.07	0.73	-0.06	0.77	0.15	0.48	-0.16	0.43
Obesity (n = 14)	-0.06	0.82	0.16	0.58	-0.15	0.59	-0.03	0.90

1 Spearman correlation coefficient.

2 Pearson correlation.

## DISCUSSION

The present study showed an agreement between TEE-ACC and TEE-DLW estimations in low-income women and different BMI classifications. According to the parameters evaluated, it was possible to observe that triaxial accelerometers could yield a modestly accurate estimate of TEE-DLW in low-income women. However, this prediction was better in participants with overweight or obesity and substantially worse in those with normal weight, with a wide range of the 95% limits of agreement, indicating poor prediction power at the individual level. Furthermore, the factorial method using the equation proposed by FAO/WHO/UNU (4) seems to produce lower bias than using data from the activPAL accelerometer.

The results of the present study were similar to those of other types of accelerometers. Brazeau and cols. (16) stated that when evaluating 22 healthy individuals aged between 18 and 45 years with a BMI between 20 and 30 kg/m^2^, in which 15% of the sample was overweight, the Actical^®^ and SenseWear Armband activity monitors when compared to the TEE-DLW showed biases of -244 ± 258 kcal/day and 94 ± 319 kcal/day, respectively. Similar results were found when evaluating individuals under different conditions, as in the study proposed by Nishida and cols. (25). When verifying the validity of triaxial accelerometers (Active style Pro HJA-750C) for estimating TEE in elderly patients (61-79y) with type 2 diabetes mellitus, they found a bias of -150 ± 183 kcal/day. In a study by Guimarães and cols. (12), when studying individuals infected with human immunodeficiency virus, the bias between the accelerometry method (activPAL^TM^) and TEE-DLW was even smaller (55 ± 485 kcal/day). In a large systematic review with meta-analysis aimed at evaluating the validity of several activity monitors, these monitors showed an underestimation in relation to the TEE-DLW (Pooled Hedges’ g: -0.68, 95% CI: - 1.15; -0.21; n = 16; p = 0.005) (10). It is worth noting that none of these studies aimed to assess the differences in prediction by BMI category.

Interestingly, the results of the present study show that individuals with normal weight have a large bias between the methods (-417 kcal/day; -21%), while participants with overweight and obesity had almost the same modular difference and drastically lower values between the methods (approximately ± 100 kcal/day; ± 4%). This difference in bias between the BMI classifications is corroborated by the positive correlation of the bias, and BMI is presented as a continuous variable. activPAL^TM^ accelerometers have already been validated for the measurement of low-intensity physical activities, such as those related to sitting, standing, and sedentary behavior (26-28). However, these accelerometers are not the most adequate for measuring moderate-to-vigorous intensity activities (29,30). This may be indicative of the finding of a greater underestimation of TEE-ACC in relation to TEE-DLW in individuals with normal weight since, possibly, they would perform more activities of moderate to vigorous intensity (31), which could increase the TEE-DLW but would not be adequately measured by the accelerometers used.

An important fact is highlighted regarding the use of the MET as a means to calculate the TEE-ACC, which can generate discrepancies when compared to TEE-DLW. According to Ainsworth and cols. (22), one MET corresponds to approximately 3.5 mL of oxygen/kg/min or 1 kcal/kg/h, and this metric is used for several measurements of energy expenditure in activities; however, these values do not seem possible to be applied to all individuals, due to different oxygen consumption, which can lead to inaccuracies in the TEE estimation (32,33). Such a measurement could become more accurate if measurements of oxygen consumption were performed by indirect calorimetry, thus adjusting to what would correspond to one MET, as indicated in the study by Macena and cols. (34). Further studies evaluating how this established oxygen consumption pattern may be in disagreement should be carried out, as shown in a systematic review, in which it was suggested that in the absence of indirect calorimetry, the value of one MET should correspond to 2.7 mL of oxygen/kg/min for adults aged 60 years or older (35).

Another fact that should be emphasized is that the TEE calculation using the factorial method (BMR calculated by predictive equations x PAL) improved the systematic error, especially in the group of women with normal weight, which is corroborated by the moderate correlation between the bias and body weight. Despite the already shown various criticisms on the calculation of energy expenditure using predictive equations, the factorial method (BMR x PAL) may probably prove to be more adequate than the use of multiples of body weight, as done in the present study (PAL x body weight x 24h), to calculate the TEE. This improvement in the systematic error may be due to the lower magnitude given to body weight for the TEE calculation, as already partially discussed (36), however, we do not have enough data to carry out further analysis in this aspect.

The present study had some limitations. First, we highlight the lack of oxygen consumption data for each participant so that the transformation from MET to TEE could be performed individually. However, the present study was designed to verify the TEE estimation capacity of triaxial accelerometers and compare them with the DLW method, for which we used the data as recommended by the manufacturer, who suggested using the MET value as established by Ainsworth and cols. (22). Another possible limitation of the present study is that PAL and MET were used synonymously, however, the former is a multiple of the BMR, while the latter is a multiple of the resting metabolic rate. Although conceptual differences may reflect differences between 10 and 20% (37), these two terms (basal and resting metabolic rate) are often used interchangeably in the literature, sometimes making their differentiation difficult. Finally, we highlight the lack of qualitative data on the physical activities performed by the participants while using the accelerometers, which could help in the classification of light, moderate or intense physical activities and, later, could enrich the performance of sensitivity analyses.

Finally, it can be concluded that the activPAL^®^ triaxial accelerometers produced reasonable estimates in relation to the TEE-DLW, mainly in low-income overweight and obese women, and were much less accurate in individuals with normal weight, and may be useful in epidemiological research proposals but not useful for individual analyzes due to the wide 95% limits of agreement, which indicate poor prediction performance at the individual level. Finally, we emphasize that it may be interesting to include individualized measurements of BMR by indirect calorimetry or even by predictive equations to complement TEE estimates using accelerometers to reduce discrepancies between the methods.
